# Bis{tris­[3-(2-pyrid­yl)-1*H*-pyrazole]zinc(II)} dodeca­molybdosilicate hexa­hydrate

**DOI:** 10.1107/S160053681000019X

**Published:** 2010-01-09

**Authors:** Xiutang Zhang, Peihai Wei, Wencai Zhu, Bin Li, Bo Hu

**Affiliations:** a Advanced Material Institute of Research, Department of Chemistry and Chemical Engineering, ShanDong Institute of Education, Jinan 250013, People’s Republic of China; b College of Chemistry and Chemical Engineering, Liaocheng University, Liaocheng 252059, People’s Republic of China

## Abstract

The asymmetric unit of the title compound, [Zn(C_8_H_7_N_3_)_3_]_2_[SiMo_12_O_40_]·6H_2_O, consists of a complex [Zn(C_8_H_7_N_3_)_3_]^2+^ cation, half of a Keggin-type [SiMo_12_O_40_]^4−^ heteropolyanion and three uncoordinated water mol­ecules. The Zn^2+^ cation is surrounded in a distorted octa­hedral coordination by six N atoms from three chelating 3-(2-pyrid­yl)pyrazole ligands. In the heteropolyanion, two O atoms of the central SiO_4_ group (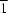 symmetry) are equally disordered about an inversion centre. N—H⋯O hydrogen bonding between the cations, anions and the uncoordinated water mol­ecules leads to a consolidation of the structure.

## Related literature

For general background to polyoxometalates, see: Pope & Müller (1991 ▶). For polyoxometalates modified with amines, see: Zhang, Dou *et al.* (2009 ▶); Zhang, Wei *et al.* (2009 ▶). For the structure of another dodeca­molybdosilicate, see: Wu *et al.* (2003 ▶).
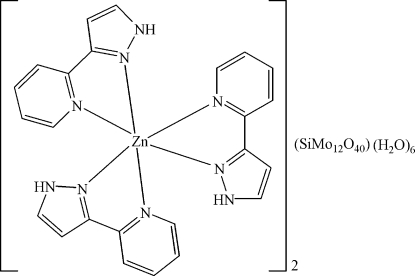


## Experimental

### 

#### Crystal data

[Zn(C_8_H_7_N_3_)_3_]_2_[SiMo_12_O_40_]·6H_2_O
*M* *_r_* = 2929.20
Monoclinic, 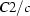

*a* = 18.824 (4) Å
*b* = 16.365 (3) Å
*c* = 27.749 (6) Å
β = 104.74 (3)°
*V* = 8267 (3) Å^3^
*Z* = 4
Mo *K*α radiation
μ = 2.44 mm^−1^
*T* = 293 K
0.12 × 0.10 × 0.08 mm

#### Data collection

Bruker APEXII CCD diffractometer
Absorption correction: multi-scan (*SADABS*; Bruker, 2001 ▶) *T*_min_ = 0.758, *T*_max_ = 0.829
27894 measured reflections
7081 independent reflections
5632 reflections with *I* > 2σ(*I*)’
*R*_int_ = 0.050

#### Refinement

*R*[*F* ^2^ > 2σ(*F* ^2^)] = 0.049
*wR*(*F* ^2^) = 0.112
*S* = 1.00
7081 reflections
601 parameters
H-atom parameters constrained
Δρ_max_ = 1.22 e Å^−3^
Δρ_min_ = −0.63 e Å^−3^

### 

Data collection: *APEX2* (Bruker, 2004 ▶); cell refinement: *SAINT-Plus* (Bruker, 2001 ▶); data reduction: *SAINT-Plus*; program(s) used to solve structure: *SHELXS97* (Sheldrick, 2008 ▶); program(s) used to refine structure: *SHELXL97* (Sheldrick, 2008 ▶); molecular graphics: *XP* in *SHELXTL* (Sheldrick, 2008 ▶); software used to prepare material for publication: *SHELXTL*.

## Supplementary Material

Crystal structure: contains datablocks global, I. DOI: 10.1107/S160053681000019X/wm2292sup1.cif

Structure factors: contains datablocks I. DOI: 10.1107/S160053681000019X/wm2292Isup2.hkl

Additional supplementary materials:  crystallographic information; 3D view; checkCIF report

## Figures and Tables

**Table 1:** Selected bond lengths (Å)

Si1—O5*A*	1.581 (8)
Si1—O20*B*	1.628 (8)
Si1—O5*B*	1.650 (9)
Si1—O20*A*	1.674 (8)
Zn1—N5	2.134 (7)
Zn1—N2	2.138 (8)
Zn1—N7	2.167 (6)
Zn1—N1	2.176 (8)
Zn1—N8	2.186 (7)
Zn1—N4	2.196 (7)

**Table 2:** Hydrogen-bond geometry (Å, °)

*D*—H⋯*A*	*D*—H	H⋯*A*	*D*⋯*A*	*D*—H⋯*A*
N9—H9*A*⋯O16*A*^i^	0.86	2.20	2.972 (14)	149
N9—H9*A*⋯O16*B*^i^	0.86	1.88	2.728 (13)	168
N6—H6⋯O2*W*	0.86	1.95	2.770 (11)	160
N3—H3*A*⋯O3*W*	0.86	2.02	2.870 (15)	167

Symmetry code: (i) 
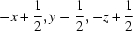
.

**Fig. 1.**
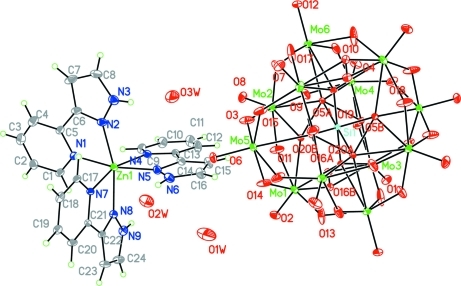
The building blocks of the title compound with the atom-labelling scheme. Displacement ellipsoids are drawn at the 30% probability level; H atoms are given as spheres of arbitrary radius.

**Fig. 2.**
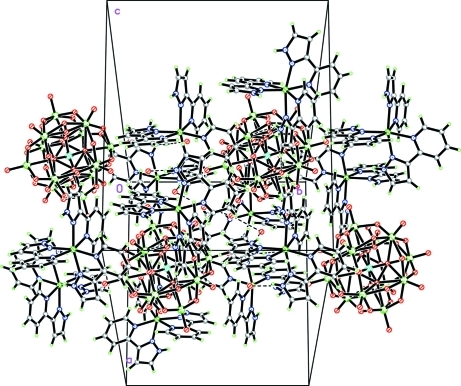
The crystal packing of the title compound, displayed with N—H···O and O—H···O hydrogen bonds as dashed lines.

## supplementary crystallographic information

### Comment

The design and synthesis of polyoxometalates has attracted continuous research interest not only because of their appealing structural and topological novelties, but also due to their interesting optical, electronic, magnetic, and catalytic properties, as well as their potential medical applications (Pope & Müller, 1991). In our research group, organic amines, such as 3-(2-pyridyl)pyrazole and pyrazine, are used to effectively modify polyoxomolybdates under hydrothermal condictions (Zhang, Dou *et al.*, 2009; Zhang, Wei *et al.*, 2009). Here, we describe the synthesis and structural characterization of the title compound.

As shown in Figure 1, the title compound consists of three subunits, *viz.* of a complex [Zn(C_8_H_7_N_3_)_3_]^2+^ cation, a heteropolyanion [SiMo_12_O_40_]^4-^ anion and of uncoordinated water molecules. The zinc(II) ion is in a distorted octahedral coordination by six N atoms from three chelating 3-(2-pyridyl)pyrazole ligands. The Zn—N bond lengths are in the range of 2.134 (7)—2.196 (7) Å. In the Keggin-type heteropolyanion, each Mo atom is surrounded by six O atoms and the Si atom is located at the center of the anion. There are four kinds of O atoms present in the anion according to their coordination environments: O*_a_* (O atoms in the disordered SiO_4_ tetrahedron), O*_b_* (bridging O atoms between two triplet groups of MoO_6_ octahedra), O*_c_* (bridging O atoms within one triplet group of MoO_6_ octahedra) and O*_d_* (terminal O atoms). The Si—O bond distances are in the normal range of 1.581 (8)—1.674 (8) compared to reported distances in other dodecamolybdosilicates (Wu *et al.*, 2003). The Mo—O bond distances vary widely from 1.647 (5) to 2.447 (8) Å. The shortest Mo—O bonds are in the range of 1.647 (5)—1.675 (5) Å for the terminal oxygen atoms. The longest Mo—O lengths are in the range of 2.345 (8)—2.447 (8) Å for those oxygen atoms connected with both Mo and Si atoms.

N—H···O and O—H···O hydrogen bonding between the cationic and anionic moieties and the uncoordinated water molecules leads to a consolidation of the structure (Fig. 2; Table 2).

### Experimental

A mixture of 3-(2-pyridyl)pyrazole (1 mmoL 0.14 g), sodium molybdate (2 mmoL, 0.48 g), sodium silicate nonahydrate (0.2 mmoL, 0.05 g) and zinc acetate (1 mmoL, 0.18 g) in 10 ml distilled water was sealed in a 25 ml Teflon-lined stainless steel autoclave and was kept at 433 K for three days. Colorless crystals suitable for the X-ray experiment were obtained. Anal. Calc. for C_48_H_54_Mo_12_N_18_O_46_SiZn_2_: C 19.67, H 1.84, N 8.60 %; Found: C 19.52, H 1.74, N 8.48 %.

### Refinement

All hydrogen atoms bound to aromatic carbon atoms were refined in calculated positions using a riding model with a C—H distance of 0.93 Å and *U*_iso_ = 1.2*U*_eq_(C). Hydrogen atoms attached to aromatic N atoms were refined with a N—H distance of 0.86 Å and *U*_iso_ = 1.2*U*_eq_(N). The hydrogen atoms of the three uncoordinated water molecules could not be located unambiguously from difference Fourier maps, probably due to disorder of the water molecules. Thus the structure was refined without the H atoms of the water molecules (which includes the water O atoms O1W, O2W, O3W). In the SiO_4_ unit, the two oxygen atoms (O5 and O20) are equally disordered about the inversion centre. One of the bridging O atoms (O16) is also disordered and was refined with split positions and an occupancy ratio of 1:1. In the final difference Fourier map the highest peak is 2.93 Å from atom O3w and the deepest hole is 0.20 A Å from atom O17. The highest peak is located in the voids of the crystal structure and may be associated with an additional water molecule. However, refinement of this position did not result in a reasonable model. Hence this position was also excluded from the final refinement.

### Crystal data

**Table:**

[Zn(C_8_H_7_N_3_)_3_]_2_[SiMo_12_O_40_]·6H_2_O	*F*(000) = 5656
*M**_r_* = 2929.20	*D*_x_ = 2.354 Mg m^−^^3^
Monoclinic, *C*2/*c*	Mo *K*α radiation, λ = 0.71073 Å
Hall symbol: -C 2yc	Cell parameters from 7081 reflections
*a* = 18.824 (4) Å	θ = 2.1–25.0°
*b* = 16.365 (3) Å	µ = 2.44 mm^−^^1^
*c* = 27.749 (6) Å	*T* = 293 K
β = 104.74 (3)°	Block, colorless
*V* = 8267 (3) Å^3^	0.12 × 0.10 × 0.08 mm
*Z* = 4	

### Data collection

**Table:**

Bruker APEXII CCD diffractometer	7081 independent reflections
Radiation source: fine-focus sealed tube	5632 reflections with *I* > 2σ(*I*)'
graphite	*R*_int_ = 0.050
φ and ω scans	θ_max_ = 25.0°, θ_min_ = 1.7°
Absorption correction: multi-scan (*SADABS*; Bruker, 2001)	*h* = −22→22
*T*_min_ = 0.758, *T*_max_ = 0.829	*k* = −19→19
27894 measured reflections	*l* = −32→32

### Refinement

**Table:**

Refinement on *F*^2^	Primary atom site location: structure-invariant direct methods
Least-squares matrix: full	Secondary atom site location: difference Fourier map
*R*[*F*^2^ > 2σ(*F*^2^)] = 0.049	Hydrogen site location: inferred from neighbouring sites
*wR*(*F*^2^) = 0.112	H-atom parameters constrained
*S* = 1.00	*w* = 1/[σ^2^(*F*_o_^2^) + (0.040*P*)^2^ + 90.4942*P*] where *P* = (*F*_o_^2^ + 2*F*_c_^2^)/3
7081 reflections	(Δ/σ)_max_ = 0.001
601 parameters	Δρ_max_ = 1.22 e Å^−^^3^
0 restraints	Δρ_min_ = −0.63 e Å^−^^3^

### Special details

**Table:**

Geometry. All esds (except the esd in the dihedral angle between two l.s. planes) are estimated using the full covariance matrix. The cell esds are taken into account individually in the estimation of esds in distances, angles and torsion angles; correlations between esds in cell parameters are only used when they are defined by crystal symmetry. An approximate (isotropic) treatment of cell esds is used for estimating esds involving l.s. planes.
Refinement. Refinement of F^2^ against ALL reflections. The weighted R-factor wR and goodness of fit S are based on F^2^, conventional R-factors R are based on F, with F set to zero for negative F^2^. The threshold expression of F^2^ > 2sigma(F^2^) is used only for calculating R-factors(gt) etc. and is not relevant to the choice of reflections for refinement. R-factors based on F^2^ are statistically about twice as large as those based on F, and R- factors based on ALL data will be even larger.

### Fractional atomic coordinates and isotropic or equivalent isotropic displacement parameters (Å^2^)

**Table:**

	*x*	*y*	*z*	*U*_iso_*/*U*_eq_	Occ. (<1)
Si1	0.2500	0.7500	0.5000	0.0259 (5)	
Zn1	0.20485 (5)	0.18036 (6)	0.30659 (4)	0.0495 (2)	
Mo1	0.32661 (4)	0.65322 (4)	0.41132 (2)	0.04445 (19)	
Mo2	0.14843 (3)	0.59791 (4)	0.42128 (2)	0.04067 (18)	
Mo3	0.25254 (4)	0.85217 (4)	0.38792 (2)	0.0493 (2)	
Mo4	0.08217 (3)	0.80182 (4)	0.41417 (3)	0.04398 (19)	
Mo5	0.30421 (4)	0.54390 (4)	0.51646 (3)	0.04711 (19)	
Mo6	0.08169 (3)	0.67952 (4)	0.51953 (3)	0.04175 (18)	
C1	0.1721 (5)	0.0214 (6)	0.2386 (4)	0.062 (2)	
H1	0.2038	0.0432	0.2211	0.074*	
C2	0.1437 (6)	−0.0550 (6)	0.2264 (4)	0.071 (3)	
H2	0.1555	−0.0844	0.2008	0.085*	
C3	0.0987 (7)	−0.0867 (8)	0.2518 (5)	0.092 (4)	
H3	0.0796	−0.1389	0.2440	0.111*	
C4	0.0811 (6)	−0.0452 (8)	0.2878 (5)	0.081 (3)	
H4	0.0498	−0.0673	0.3055	0.097*	
C5	0.1100 (4)	0.0311 (6)	0.2985 (3)	0.057 (2)	
C6	0.0921 (5)	0.0815 (7)	0.3375 (4)	0.065 (3)	
C7	0.0418 (5)	0.0710 (10)	0.3689 (5)	0.100 (5)	
H7	0.0105	0.0273	0.3694	0.120*	
C8	0.0516 (6)	0.1423 (9)	0.3984 (5)	0.084 (4)	
H8	0.0269	0.1557	0.4224	0.101*	
C9	0.0912 (5)	0.2438 (6)	0.2080 (4)	0.068 (3)	
H9	0.0867	0.1889	0.1989	0.082*	
C10	0.0527 (6)	0.2998 (8)	0.1755 (5)	0.089 (4)	
H10	0.0228	0.2832	0.1450	0.107*	
C11	0.0582 (6)	0.3805 (8)	0.1880 (4)	0.092 (4)	
H11	0.0320	0.4197	0.1665	0.110*	
C12	0.1037 (6)	0.4026 (7)	0.2336 (4)	0.086 (3)	
H12	0.1083	0.4571	0.2434	0.103*	
C13	0.1420 (5)	0.3430 (5)	0.2640 (3)	0.055 (2)	
C14	0.1945 (5)	0.3607 (6)	0.3124 (3)	0.057 (2)	
C15	0.2158 (6)	0.4342 (6)	0.3378 (4)	0.073 (3)	
H15	0.1974	0.4861	0.3280	0.088*	
C16	0.2680 (6)	0.4148 (7)	0.3790 (4)	0.074 (3)	
H16	0.2928	0.4508	0.4035	0.089*	
C17	0.2987 (4)	0.1008 (5)	0.4058 (3)	0.0471 (19)	
H17	0.2562	0.1054	0.4169	0.057*	
C18	0.3603 (5)	0.0661 (5)	0.4374 (3)	0.056 (2)	
H18	0.3585	0.0477	0.4687	0.067*	
C19	0.4222 (5)	0.0591 (5)	0.4225 (3)	0.053 (2)	
H19	0.4639	0.0357	0.4433	0.063*	
C20	0.4231 (4)	0.0872 (6)	0.3760 (3)	0.054 (2)	
H20	0.4659	0.0835	0.3652	0.065*	
C21	0.3604 (4)	0.1210 (4)	0.3454 (3)	0.0397 (17)	
C22	0.3555 (4)	0.1495 (5)	0.2946 (3)	0.0416 (18)	
C23	0.4101 (5)	0.1560 (7)	0.2682 (3)	0.067 (3)	
H23	0.4597	0.1429	0.2794	0.080*	
C24	0.3738 (6)	0.1861 (7)	0.2224 (4)	0.076 (3)	
H24	0.3941	0.1963	0.1957	0.091*	
N1	0.1557 (4)	0.0657 (4)	0.2749 (3)	0.0557 (18)	
N2	0.1281 (4)	0.1523 (5)	0.3493 (3)	0.062 (2)	
N3	0.1028 (5)	0.1869 (6)	0.3854 (3)	0.076 (2)	
H3A	0.1181	0.2331	0.3988	0.091*	
N4	0.1355 (4)	0.2636 (5)	0.2524 (3)	0.0543 (18)	
N5	0.2323 (4)	0.2991 (4)	0.3376 (3)	0.0559 (18)	
N6	0.2775 (4)	0.3336 (5)	0.3781 (3)	0.066 (2)	
H6	0.3087	0.3068	0.4006	0.079*	
N7	0.2976 (3)	0.1278 (4)	0.3605 (2)	0.0394 (14)	
N8	0.2916 (4)	0.1760 (4)	0.2672 (2)	0.0467 (16)	
N9	0.3042 (4)	0.1979 (5)	0.2231 (2)	0.061 (2)	
H9A	0.2710	0.2171	0.1984	0.073*	
O1	0.2556 (3)	0.9034 (4)	0.3374 (2)	0.0581 (15)	
O2	0.3551 (3)	0.6067 (4)	0.3660 (2)	0.0603 (16)	
O3	0.2143 (3)	0.5308 (5)	0.4729 (3)	0.092 (2)	
O4	0.0056 (3)	0.8237 (4)	0.3718 (2)	0.0709 (19)	
O5A	0.1705 (4)	0.7185 (5)	0.4728 (3)	0.0272 (19)	0.50
O5B	0.2051 (5)	0.8171 (5)	0.4590 (3)	0.029 (2)	0.50
O6	0.3318 (3)	0.4476 (3)	0.5234 (3)	0.0649 (17)	
O7	0.0948 (4)	0.6044 (4)	0.4756 (2)	0.0711 (19)	
O8	0.0983 (4)	0.5235 (4)	0.3883 (2)	0.0671 (18)	
O9	0.0955 (4)	0.6878 (4)	0.3938 (2)	0.0730 (19)	
O10	0.0468 (4)	0.7568 (4)	0.4653 (2)	0.0698 (19)	
O11	0.2221 (3)	0.6113 (5)	0.3913 (2)	0.085 (2)	
O12	0.0004 (3)	0.6534 (4)	0.5292 (3)	0.0649 (17)	
O13	0.4022 (5)	0.7155 (4)	0.4407 (3)	0.113 (4)	
O14	0.3436 (3)	0.5746 (5)	0.4594 (3)	0.092 (3)	
O15	0.2669 (4)	0.5588 (5)	0.5708 (3)	0.113 (3)	
O16A	0.2666 (7)	0.7439 (8)	0.3817 (5)	0.042 (3)	0.50
O16B	0.2893 (7)	0.7499 (7)	0.3635 (4)	0.034 (3)	0.50
O17	0.1467 (5)	0.6329 (4)	0.5734 (3)	0.112 (3)	
O18	0.1061 (4)	0.9000 (5)	0.4463 (3)	0.100 (3)	
O19	0.1530 (4)	0.8255 (4)	0.3747 (3)	0.089 (2)	
O20A	0.3000 (4)	0.7634 (5)	0.4586 (3)	0.0257 (19)	0.50
O20B	0.2524 (5)	0.6636 (5)	0.4715 (3)	0.030 (2)	0.50
O1W	0.4635 (8)	0.4314 (11)	0.4744 (9)	0.278 (10)	
O2W	0.3838 (4)	0.2836 (6)	0.4615 (3)	0.109 (3)	
O3W	0.1316 (7)	0.3429 (7)	0.4336 (5)	0.159 (5)	

### Atomic displacement parameters (Å^2^)

**Table:**

	*U*^11^	*U*^22^	*U*^33^	*U*^12^	*U*^13^	*U*^23^
Si1	0.0238 (12)	0.0280 (13)	0.0247 (13)	0.0012 (10)	0.0038 (10)	−0.0011 (10)
Zn1	0.0419 (5)	0.0506 (6)	0.0561 (6)	0.0072 (4)	0.0128 (4)	0.0112 (5)
Mo1	0.0470 (4)	0.0519 (4)	0.0337 (4)	0.0140 (3)	0.0089 (3)	−0.0095 (3)
Mo2	0.0329 (3)	0.0450 (4)	0.0421 (4)	−0.0093 (3)	0.0058 (3)	−0.0105 (3)
Mo3	0.0725 (5)	0.0434 (4)	0.0306 (3)	−0.0072 (4)	0.0108 (3)	0.0056 (3)
Mo4	0.0293 (3)	0.0542 (4)	0.0425 (4)	0.0003 (3)	−0.0017 (3)	0.0035 (3)
Mo5	0.0524 (4)	0.0308 (4)	0.0637 (5)	0.0091 (3)	0.0250 (4)	0.0063 (3)
Mo6	0.0275 (3)	0.0455 (4)	0.0520 (4)	−0.0075 (3)	0.0095 (3)	−0.0032 (3)
C1	0.057 (6)	0.067 (6)	0.063 (6)	−0.005 (5)	0.017 (5)	0.016 (5)
C2	0.068 (6)	0.060 (6)	0.076 (7)	−0.002 (5)	0.003 (5)	−0.004 (5)
C3	0.074 (8)	0.079 (8)	0.105 (10)	−0.009 (6)	−0.016 (7)	0.012 (8)
C4	0.054 (6)	0.092 (9)	0.083 (8)	−0.020 (6)	−0.008 (6)	0.015 (7)
C5	0.033 (4)	0.063 (6)	0.066 (6)	−0.005 (4)	−0.005 (4)	0.020 (5)
C6	0.034 (4)	0.092 (8)	0.067 (6)	0.011 (5)	0.010 (4)	0.035 (6)
C7	0.042 (6)	0.140 (12)	0.115 (10)	0.002 (7)	0.014 (6)	0.076 (10)
C8	0.062 (7)	0.120 (10)	0.079 (8)	0.032 (7)	0.034 (6)	0.040 (8)
C9	0.068 (6)	0.066 (6)	0.061 (6)	−0.009 (5)	0.000 (5)	0.008 (5)
C10	0.081 (8)	0.088 (9)	0.084 (8)	0.004 (7)	−0.007 (6)	0.022 (7)
C11	0.084 (8)	0.083 (9)	0.086 (8)	0.019 (6)	−0.018 (7)	0.027 (7)
C12	0.087 (8)	0.059 (6)	0.097 (9)	0.017 (6)	−0.005 (7)	0.016 (6)
C13	0.051 (5)	0.052 (5)	0.063 (6)	0.008 (4)	0.013 (4)	0.006 (4)
C14	0.058 (5)	0.059 (6)	0.057 (5)	0.013 (4)	0.019 (4)	0.001 (5)
C15	0.099 (8)	0.061 (6)	0.063 (6)	0.021 (6)	0.025 (6)	−0.002 (5)
C16	0.100 (8)	0.072 (7)	0.053 (6)	0.008 (6)	0.025 (6)	−0.013 (5)
C17	0.043 (4)	0.050 (5)	0.051 (5)	−0.004 (4)	0.018 (4)	0.010 (4)
C18	0.073 (6)	0.059 (5)	0.029 (4)	−0.015 (5)	0.002 (4)	0.013 (4)
C19	0.045 (5)	0.058 (5)	0.047 (5)	−0.007 (4)	−0.001 (4)	0.009 (4)
C20	0.027 (4)	0.077 (6)	0.054 (5)	−0.001 (4)	0.002 (4)	0.012 (4)
C21	0.036 (4)	0.037 (4)	0.047 (4)	0.003 (3)	0.012 (3)	−0.001 (3)
C22	0.039 (4)	0.046 (5)	0.039 (4)	−0.004 (3)	0.011 (3)	0.010 (3)
C23	0.041 (5)	0.109 (8)	0.056 (6)	0.009 (5)	0.022 (4)	0.011 (5)
C24	0.068 (7)	0.114 (9)	0.055 (6)	−0.007 (6)	0.030 (5)	0.000 (6)
N1	0.044 (4)	0.053 (4)	0.068 (5)	0.001 (3)	0.009 (4)	0.015 (4)
N2	0.046 (4)	0.079 (6)	0.063 (5)	0.015 (4)	0.017 (4)	0.023 (4)
N3	0.066 (5)	0.083 (6)	0.080 (6)	0.023 (5)	0.019 (5)	0.021 (5)
N4	0.044 (4)	0.062 (5)	0.057 (5)	0.007 (3)	0.014 (3)	0.006 (4)
N5	0.049 (4)	0.053 (4)	0.062 (5)	0.007 (3)	0.008 (4)	0.002 (4)
N6	0.065 (5)	0.077 (6)	0.053 (5)	0.019 (4)	0.012 (4)	0.005 (4)
N7	0.036 (3)	0.045 (4)	0.039 (3)	0.007 (3)	0.011 (3)	0.009 (3)
N8	0.046 (4)	0.055 (4)	0.037 (4)	0.004 (3)	0.007 (3)	0.005 (3)
N9	0.075 (5)	0.071 (5)	0.033 (4)	0.002 (4)	0.007 (3)	0.016 (3)
O1	0.061 (4)	0.072 (4)	0.039 (3)	−0.012 (3)	0.007 (3)	0.013 (3)
O2	0.052 (3)	0.085 (4)	0.046 (3)	0.001 (3)	0.016 (3)	−0.028 (3)
O3	0.047 (3)	0.152 (6)	0.083 (4)	0.019 (4)	0.029 (3)	0.065 (4)
O4	0.052 (4)	0.102 (5)	0.049 (4)	0.031 (4)	−0.006 (3)	0.004 (3)
O5A	0.024 (4)	0.027 (5)	0.031 (5)	−0.001 (4)	0.009 (4)	−0.003 (4)
O5B	0.029 (5)	0.030 (5)	0.029 (5)	−0.004 (4)	0.005 (4)	−0.001 (4)
O6	0.062 (4)	0.030 (3)	0.103 (5)	0.013 (3)	0.020 (3)	0.011 (3)
O7	0.112 (5)	0.052 (4)	0.061 (4)	0.038 (4)	0.044 (4)	0.015 (3)
O8	0.083 (5)	0.054 (4)	0.060 (4)	−0.025 (3)	0.011 (3)	−0.021 (3)
O9	0.114 (6)	0.053 (4)	0.068 (4)	0.029 (4)	0.053 (4)	0.010 (3)
O10	0.119 (6)	0.051 (4)	0.054 (4)	0.032 (4)	0.048 (4)	0.012 (3)
O11	0.039 (3)	0.144 (7)	0.073 (4)	0.009 (4)	0.016 (3)	0.060 (5)
O12	0.050 (3)	0.063 (4)	0.094 (5)	−0.004 (3)	0.043 (3)	0.003 (3)
O13	0.131 (7)	0.035 (4)	0.113 (6)	0.021 (4)	−0.080 (5)	−0.017 (4)
O14	0.026 (3)	0.175 (8)	0.074 (4)	0.008 (4)	0.010 (3)	0.070 (5)
O15	0.102 (6)	0.123 (6)	0.147 (7)	−0.079 (5)	0.095 (6)	−0.093 (6)
O16A	0.038 (8)	0.056 (8)	0.030 (7)	−0.004 (6)	0.008 (5)	0.002 (6)
O16B	0.037 (7)	0.038 (6)	0.024 (7)	−0.002 (5)	0.001 (5)	−0.001 (5)
O17	0.124 (6)	0.040 (4)	0.116 (6)	0.026 (4)	−0.073 (5)	−0.025 (4)
O18	0.105 (6)	0.116 (6)	0.108 (6)	−0.068 (5)	0.080 (5)	−0.069 (5)
O19	0.093 (5)	0.088 (5)	0.108 (5)	−0.042 (4)	0.069 (4)	−0.046 (4)
O20A	0.023 (4)	0.030 (5)	0.024 (4)	−0.006 (4)	0.006 (4)	−0.006 (4)
O20B	0.034 (5)	0.031 (5)	0.027 (5)	0.000 (4)	0.012 (4)	−0.003 (4)
O1W	0.178 (14)	0.240 (17)	0.47 (3)	0.016 (12)	0.190 (18)	−0.077 (19)
O2W	0.080 (5)	0.143 (8)	0.090 (6)	0.026 (5)	−0.003 (4)	0.006 (5)
O3W	0.186 (11)	0.135 (9)	0.200 (12)	−0.027 (8)	0.128 (10)	−0.028 (8)

### Geometric parameters (Å, °)

**Table:**

Si1—O5A	1.581 (8)	C2—H2	0.9300
Si1—O5A^i^	1.581 (8)	C3—C4	1.320 (16)
Si1—O20B^i^	1.628 (8)	C3—H3	0.9300
Si1—O20B	1.628 (8)	C4—C5	1.364 (14)
Si1—O5B	1.650 (9)	C4—H4	0.9300
Si1—O5B^i^	1.650 (9)	C5—N1	1.333 (11)
Si1—O20A^i^	1.674 (8)	C5—C6	1.466 (14)
Si1—O20A	1.674 (8)	C6—N2	1.340 (12)
Zn1—N5	2.134 (7)	C6—C7	1.450 (14)
Zn1—N2	2.138 (8)	C7—C8	1.411 (18)
Zn1—N7	2.167 (6)	C7—H7	0.9300
Zn1—N1	2.176 (8)	C8—N3	1.329 (14)
Zn1—N8	2.186 (7)	C8—H8	0.9300
Zn1—N4	2.196 (7)	C9—N4	1.341 (11)
Mo1—O2	1.670 (5)	C9—C10	1.357 (14)
Mo1—O13	1.771 (7)	C9—H9	0.9300
Mo1—O14	1.823 (6)	C10—C11	1.363 (16)
Mo1—O16A	1.918 (13)	C10—H10	0.9300
Mo1—O11	2.023 (6)	C11—C12	1.382 (15)
Mo1—O16B	2.070 (12)	C11—H11	0.9300
Mo1—O20A	2.358 (8)	C12—C13	1.368 (13)
Mo1—O20B	2.439 (8)	C12—H12	0.9300
Mo2—O8	1.666 (5)	C13—N4	1.336 (11)
Mo2—O11	1.803 (6)	C13—C14	1.480 (12)
Mo2—O9	1.830 (6)	C14—N5	1.326 (11)
Mo2—O3	1.972 (6)	C14—C15	1.399 (13)
Mo2—O7	2.019 (6)	C15—C16	1.343 (14)
Mo2—O20B	2.353 (9)	C15—H15	0.9300
Mo2—O5A	2.411 (8)	C16—N6	1.342 (12)
Mo3—O1	1.647 (5)	C16—H16	0.9300
Mo3—O16A	1.806 (14)	C17—N7	1.328 (9)
Mo3—O19	1.867 (7)	C17—C18	1.383 (11)
Mo3—O17^i^	1.943 (7)	C17—H17	0.9300
Mo3—O15^i^	1.945 (7)	C18—C19	1.337 (12)
Mo3—O16B	1.994 (12)	C18—H18	0.9300
Mo3—O20A	2.423 (8)	C19—C20	1.375 (11)
Mo3—O5B	2.433 (9)	C19—H19	0.9300
Mo4—O4	1.651 (5)	C20—C21	1.382 (10)
Mo4—O18	1.837 (7)	C20—H20	0.9300
Mo4—O10	1.867 (6)	C21—N7	1.357 (9)
Mo4—O19	1.967 (6)	C21—C22	1.467 (10)
Mo4—O9	1.984 (6)	C22—N8	1.322 (9)
Mo4—O5B	2.345 (8)	C22—C23	1.410 (11)
Mo4—O5A	2.427 (8)	C23—C24	1.373 (13)
Mo5—O6	1.655 (5)	C23—H23	0.9300
Mo5—O3	1.825 (7)	C24—N9	1.331 (12)
Mo5—O15	1.834 (7)	C24—H24	0.9300
Mo5—O18^i^	1.968 (7)	N2—N3	1.339 (11)
Mo5—O14	1.975 (6)	N3—H3A	0.8600
Mo5—O20B	2.393 (9)	N5—N6	1.350 (10)
Mo5—O5B^i^	2.394 (9)	N6—H6	0.8600
Mo6—O12	1.675 (5)	N8—N9	1.351 (9)
Mo6—O7	1.792 (6)	N9—H9A	0.8600
Mo6—O17	1.839 (6)	O5A—O20B	1.794 (12)
Mo6—O10	1.947 (6)	O5A—O5B	1.816 (12)
Mo6—O13^i^	2.022 (6)	O5B—Mo5^i^	2.394 (9)
Mo6—O20A^i^	2.348 (8)	O13—Mo6^i^	2.022 (6)
Mo6—O5A	2.447 (8)	O15—Mo3^i^	1.945 (7)
C1—N1	1.340 (12)	O16A—O16B	0.747 (11)
C1—C2	1.368 (13)	O17—Mo3^i^	1.943 (7)
C1—H1	0.9300	O18—Mo5^i^	1.968 (7)
C2—C3	1.337 (16)	O20A—Mo6^i^	2.348 (8)
			
O5A—Si1—O5A^i^	180.000 (1)	O17—Mo6—O13^i^	86.1 (3)
O5A—Si1—O20B^i^	112.0 (4)	O10—Mo6—O13^i^	81.0 (3)
O5A^i^—Si1—O20B^i^	68.0 (4)	O12—Mo6—O20A^i^	154.9 (3)
O5A—Si1—O20B	68.0 (4)	O7—Mo6—O20A^i^	99.4 (3)
O5A^i^—Si1—O20B	112.0 (4)	O17—Mo6—O20A^i^	64.2 (4)
O20B^i^—Si1—O20B	180.000 (2)	O10—Mo6—O20A^i^	93.0 (3)
O5A—Si1—O5B	68.3 (4)	O13^i^—Mo6—O20A^i^	61.3 (3)
O5A^i^—Si1—O5B	111.7 (4)	O12—Mo6—O5A	157.9 (3)
O20B^i^—Si1—O5B	71.6 (4)	O7—Mo6—O5A	65.6 (3)
O20B—Si1—O5B	108.4 (4)	O17—Mo6—O5A	97.8 (4)
O5A—Si1—O5B^i^	111.7 (4)	O10—Mo6—O5A	64.1 (3)
O5A^i^—Si1—O5B^i^	68.3 (4)	O13^i^—Mo6—O5A	92.3 (4)
O20B^i^—Si1—O5B^i^	108.4 (4)	O20A^i^—Mo6—O5A	45.7 (3)
O20B—Si1—O5B^i^	71.6 (4)	N1—C1—C2	122.0 (9)
O5B—Si1—O5B^i^	180.0 (5)	N1—C1—H1	119.0
O5A—Si1—O20A^i^	69.8 (4)	C2—C1—H1	119.0
O5A^i^—Si1—O20A^i^	110.2 (4)	C3—C2—C1	119.0 (11)
O20B^i^—Si1—O20A^i^	71.9 (4)	C3—C2—H2	120.5
O20B—Si1—O20A^i^	108.1 (4)	C1—C2—H2	120.5
O5B—Si1—O20A^i^	106.3 (4)	C2—C3—C4	121.0 (12)
O5B^i^—Si1—O20A^i^	73.7 (4)	C2—C3—H3	119.5
O5A—Si1—O20A	110.2 (4)	C4—C3—H3	119.5
O5A^i^—Si1—O20A	69.8 (4)	C3—C4—C5	118.2 (12)
O20B^i^—Si1—O20A	108.1 (4)	C3—C4—H4	120.9
O20B—Si1—O20A	71.9 (4)	C5—C4—H4	120.9
O5B—Si1—O20A	73.7 (4)	N1—C5—C4	123.6 (11)
O5B^i^—Si1—O20A	106.3 (4)	N1—C5—C6	115.0 (8)
O20A^i^—Si1—O20A	180.000 (2)	C4—C5—C6	121.4 (10)
N5—Zn1—N2	95.8 (3)	N2—C6—C7	108.9 (11)
N5—Zn1—N7	90.8 (3)	N2—C6—C5	118.0 (8)
N2—Zn1—N7	94.1 (2)	C7—C6—C5	133.1 (12)
N5—Zn1—N1	169.1 (3)	C8—C7—C6	103.9 (11)
N2—Zn1—N1	76.2 (3)	C8—C7—H7	128.0
N7—Zn1—N1	97.0 (2)	C6—C7—H7	128.0
N5—Zn1—N8	95.5 (3)	N3—C8—C7	107.0 (10)
N2—Zn1—N8	165.1 (3)	N3—C8—H8	126.5
N7—Zn1—N8	76.1 (2)	C7—C8—H8	126.5
N1—Zn1—N8	93.7 (3)	N4—C9—C10	123.2 (10)
N5—Zn1—N4	75.6 (3)	N4—C9—H9	118.4
N2—Zn1—N4	98.3 (3)	C10—C9—H9	118.4
N7—Zn1—N4	162.4 (3)	C9—C10—C11	119.5 (11)
N1—Zn1—N4	98.1 (3)	C9—C10—H10	120.2
N8—Zn1—N4	93.8 (2)	C11—C10—H10	120.3
O2—Mo1—O13	103.6 (4)	C10—C11—C12	118.4 (10)
O2—Mo1—O14	101.5 (4)	C10—C11—H11	120.8
O13—Mo1—O14	95.7 (3)	C12—C11—H11	120.8
O2—Mo1—O16A	107.3 (4)	C13—C12—C11	118.9 (11)
O13—Mo1—O16A	93.9 (4)	C13—C12—H12	120.5
O14—Mo1—O16A	146.5 (4)	C11—C12—H12	120.5
O2—Mo1—O11	96.6 (3)	N4—C13—C12	122.9 (9)
O13—Mo1—O11	158.8 (4)	N4—C13—C14	114.1 (8)
O14—Mo1—O11	86.5 (3)	C12—C13—C14	123.0 (9)
O16A—Mo1—O11	73.7 (4)	N5—C14—C15	110.0 (8)
O2—Mo1—O16B	89.3 (4)	N5—C14—C13	118.3 (8)
O13—Mo1—O16B	87.5 (4)	C15—C14—C13	131.6 (9)
O14—Mo1—O16B	167.7 (4)	C16—C15—C14	106.2 (9)
O16A—Mo1—O16B	21.2 (3)	C16—C15—H15	126.9
O11—Mo1—O16B	86.3 (4)	C14—C15—H15	126.9
O2—Mo1—O20A	157.2 (3)	C15—C16—N6	107.0 (9)
O13—Mo1—O20A	64.1 (4)	C15—C16—H16	126.5
O14—Mo1—O20A	98.9 (3)	N6—C16—H16	126.5
O16A—Mo1—O20A	57.4 (4)	N7—C17—C18	122.9 (7)
O11—Mo1—O20A	94.8 (3)	N7—C17—H17	118.5
O16B—Mo1—O20A	71.8 (4)	C18—C17—H17	118.5
O2—Mo1—O20B	153.6 (3)	C19—C18—C17	119.8 (8)
O13—Mo1—O20B	100.2 (4)	C19—C18—H18	120.1
O14—Mo1—O20B	64.7 (3)	C17—C18—H18	120.1
O16A—Mo1—O20B	82.1 (4)	C18—C19—C20	118.7 (8)
O11—Mo1—O20B	61.7 (3)	C18—C19—H19	120.7
O16B—Mo1—O20B	103.1 (4)	C20—C19—H19	120.7
O20A—Mo1—O20B	47.6 (3)	C19—C20—C21	119.9 (8)
O8—Mo2—O11	103.3 (4)	C19—C20—H20	120.0
O8—Mo2—O9	100.7 (3)	C21—C20—H20	120.1
O11—Mo2—O9	96.4 (3)	N7—C21—C20	121.2 (7)
O8—Mo2—O3	99.0 (4)	N7—C21—C22	115.1 (6)
O11—Mo2—O3	89.2 (3)	C20—C21—C22	123.6 (7)
O9—Mo2—O3	157.6 (4)	N8—C22—C23	110.8 (7)
O8—Mo2—O7	97.3 (3)	N8—C22—C21	118.9 (7)
O11—Mo2—O7	158.0 (3)	C23—C22—C21	130.3 (7)
O9—Mo2—O7	86.9 (2)	C24—C23—C22	104.5 (8)
O3—Mo2—O7	80.2 (3)	C24—C23—H23	127.8
O8—Mo2—O20B	158.3 (3)	C22—C23—H23	127.8
O11—Mo2—O20B	66.1 (3)	N9—C24—C23	107.4 (8)
O9—Mo2—O20B	99.3 (3)	N9—C24—H24	126.3
O3—Mo2—O20B	63.3 (3)	C23—C24—H24	126.3
O7—Mo2—O20B	91.9 (3)	C5—N1—C1	116.2 (8)
O8—Mo2—O5A	155.9 (3)	C5—N1—Zn1	115.4 (7)
O11—Mo2—O5A	97.9 (3)	C1—N1—Zn1	127.9 (6)
O9—Mo2—O5A	65.3 (3)	N3—N2—C6	107.0 (8)
O3—Mo2—O5A	92.5 (3)	N3—N2—Zn1	138.0 (7)
O7—Mo2—O5A	63.7 (3)	C6—N2—Zn1	115.0 (7)
O20B—Mo2—O5A	44.2 (3)	C8—N3—N2	113.1 (10)
O1—Mo3—O16A	112.4 (5)	C8—N3—H3A	123.5
O1—Mo3—O19	101.7 (3)	N2—N3—H3A	123.5
O16A—Mo3—O19	85.2 (5)	C9—N4—C13	117.0 (8)
O1—Mo3—O17^i^	99.5 (4)	C9—N4—Zn1	127.0 (7)
O16A—Mo3—O17^i^	91.7 (4)	C13—N4—Zn1	115.9 (6)
O19—Mo3—O17^i^	158.1 (4)	C14—N5—N6	105.1 (7)
O1—Mo3—O15^i^	100.0 (4)	C14—N5—Zn1	116.0 (6)
O16A—Mo3—O15^i^	147.7 (5)	N6—N5—Zn1	138.9 (6)
O19—Mo3—O15^i^	87.8 (3)	C16—N6—N5	111.7 (8)
O17^i^—Mo3—O15^i^	83.3 (3)	C16—N6—H6	124.1
O1—Mo3—O16B	92.7 (4)	N5—N6—H6	124.2
O16A—Mo3—O16B	22.0 (4)	C17—N7—C21	117.4 (6)
O19—Mo3—O16B	98.9 (4)	C17—N7—Zn1	127.0 (5)
O17^i^—Mo3—O16B	85.3 (4)	C21—N7—Zn1	115.6 (5)
O15^i^—Mo3—O16B	164.2 (5)	C22—N8—N9	105.3 (6)
O1—Mo3—O20A	155.4 (3)	C22—N8—Zn1	114.1 (5)
O16A—Mo3—O20A	57.0 (4)	N9—N8—Zn1	140.3 (5)
O19—Mo3—O20A	99.4 (3)	C24—N9—N8	112.1 (7)
O17^i^—Mo3—O20A	61.4 (3)	C24—N9—H9A	124.0
O15^i^—Mo3—O20A	93.3 (4)	N8—N9—H9A	124.0
O16B—Mo3—O20A	71.5 (4)	Mo5—O3—Mo2	136.1 (5)
O1—Mo3—O5B	155.5 (3)	Si1—O5A—O20B	57.2 (4)
O16A—Mo3—O5B	86.5 (5)	Si1—O5A—O5B	57.6 (4)
O19—Mo3—O5B	63.0 (3)	O20B—O5A—O5B	94.8 (5)
O17^i^—Mo3—O5B	95.2 (4)	Si1—O5A—Mo2	123.3 (4)
O15^i^—Mo3—O5B	62.4 (3)	O20B—O5A—Mo2	66.2 (4)
O16B—Mo3—O5B	108.0 (4)	O5B—O5A—Mo2	128.3 (5)
O20A—Mo3—O5B	48.5 (3)	Si1—O5A—Mo4	122.7 (4)
O4—Mo4—O18	102.6 (4)	O20B—O5A—Mo4	135.5 (5)
O4—Mo4—O10	102.2 (3)	O5B—O5A—Mo4	65.3 (4)
O18—Mo4—O10	93.9 (3)	Mo2—O5A—Mo4	94.4 (3)
O4—Mo4—O19	98.9 (4)	Si1—O5A—Mo6	121.6 (4)
O18—Mo4—O19	88.9 (3)	O20B—O5A—Mo6	126.0 (5)
O10—Mo4—O19	157.6 (3)	O5B—O5A—Mo6	132.3 (5)
O4—Mo4—O9	98.7 (3)	Mo2—O5A—Mo6	93.6 (3)
O18—Mo4—O9	158.1 (4)	Mo4—O5A—Mo6	93.3 (3)
O10—Mo4—O9	86.6 (2)	Si1—O5B—O5A	54.0 (4)
O19—Mo4—O9	82.8 (3)	Si1—O5B—Mo4	123.9 (4)
O4—Mo4—O5B	156.9 (3)	O5A—O5B—Mo4	70.1 (4)
O18—Mo4—O5B	64.1 (4)	Si1—O5B—Mo5^i^	119.9 (4)
O10—Mo4—O5B	97.6 (3)	O5A—O5B—Mo5^i^	136.7 (5)
O19—Mo4—O5B	63.7 (3)	Mo4—O5B—Mo5^i^	96.8 (3)
O9—Mo4—O5B	94.1 (3)	Si1—O5B—Mo3	119.1 (4)
O4—Mo4—O5A	157.5 (3)	O5A—O5B—Mo3	127.3 (5)
O18—Mo4—O5A	97.2 (4)	Mo4—O5B—Mo3	96.4 (3)
O10—Mo4—O5A	65.5 (3)	Mo5^i^—O5B—Mo3	94.3 (3)
O19—Mo4—O5A	92.2 (3)	Mo6—O7—Mo2	136.5 (4)
O9—Mo4—O5A	63.1 (3)	Mo2—O9—Mo4	137.1 (4)
O5B—Mo4—O5A	44.7 (3)	Mo4—O10—Mo6	136.8 (4)
O6—Mo5—O3	100.1 (4)	Mo2—O11—Mo1	136.3 (4)
O6—Mo5—O15	101.9 (4)	Mo1—O13—Mo6^i^	137.0 (5)
O3—Mo5—O15	94.3 (3)	Mo1—O14—Mo5	137.3 (4)
O6—Mo5—O18^i^	100.4 (4)	Mo5—O15—Mo3^i^	138.8 (5)
O3—Mo5—O18^i^	158.4 (4)	O16B—O16A—Mo3	93.4 (18)
O15—Mo5—O18^i^	87.9 (3)	O16B—O16A—Mo1	90.9 (17)
O6—Mo5—O14	99.5 (3)	Mo3—O16A—Mo1	143.4 (7)
O3—Mo5—O14	88.4 (3)	O16A—O16B—Mo3	64.7 (16)
O15—Mo5—O14	157.6 (4)	O16A—O16B—Mo1	67.9 (16)
O18^i^—Mo5—O14	81.7 (3)	Mo3—O16B—Mo1	120.9 (6)
O6—Mo5—O20B	156.1 (3)	Mo6—O17—Mo3^i^	138.7 (5)
O3—Mo5—O20B	64.2 (3)	Mo4—O18—Mo5^i^	137.2 (5)
O15—Mo5—O20B	97.4 (4)	Mo3—O19—Mo4	136.5 (4)
O18^i^—Mo5—O20B	94.2 (4)	Si1—O20A—Mo6^i^	122.5 (4)
O14—Mo5—O20B	63.9 (3)	Si1—O20A—Mo1	121.2 (4)
O6—Mo5—O5B^i^	156.3 (3)	Mo6^i^—O20A—Mo1	97.2 (3)
O3—Mo5—O5B^i^	100.2 (3)	Si1—O20A—Mo3	118.6 (4)
O15—Mo5—O5B^i^	64.5 (3)	Mo6^i^—O20A—Mo3	95.8 (3)
O18^i^—Mo5—O5B^i^	61.5 (3)	Mo1—O20A—Mo3	95.4 (3)
O14—Mo5—O5B^i^	93.2 (3)	Si1—O20B—O5A	54.8 (4)
O20B—Mo5—O5B^i^	47.2 (3)	Si1—O20B—Mo2	124.2 (5)
O12—Mo6—O7	102.9 (3)	O5A—O20B—Mo2	69.6 (4)
O12—Mo6—O17	102.1 (4)	Si1—O20B—Mo5	121.0 (4)
O7—Mo6—O17	95.2 (3)	O5A—O20B—Mo5	129.6 (5)
O12—Mo6—O10	98.4 (3)	Mo2—O20B—Mo5	95.9 (3)
O7—Mo6—O10	89.9 (2)	Si1—O20B—Mo1	119.0 (4)
O17—Mo6—O10	157.1 (4)	O5A—O20B—Mo1	133.9 (5)
O12—Mo6—O13^i^	98.4 (4)	Mo2—O20B—Mo1	95.7 (3)
O7—Mo6—O13^i^	157.9 (4)	Mo5—O20B—Mo1	94.1 (3)

Symmetry codes: (i) −*x*+1/2, −*y*+3/2, −*z*+1.

### Hydrogen-bond geometry (Å, °)

**Table:**

*D*—H···*A*	*D*—H	H···*A*	*D*···*A*	*D*—H···*A*
N9—H9A···O16A^ii^	0.86	2.20	2.972 (14)	149
N9—H9A···O16B^ii^	0.86	1.88	2.728 (13)	168
N6—H6···O2W	0.86	1.95	2.770 (11)	160
N3—H3A···O3W	0.86	2.02	2.870 (15)	167

Symmetry codes: (ii) −*x*+1/2, *y*−1/2, −*z*+1/2.

## References

[bb1] Bruker (2001). *SAINT-Plus* and *SADABS* Bruker AXS Inc., Madison, Wisconsin, USA.

[bb2] Bruker (2004). *APEX2* Bruker AXS Inc., Madison, Wisconsin, USA.

[bb3] Pope, M. T. & Müller, A. (1991). *Angew. Chem. Int. Ed.* **30**, 34–38.

[bb4] Sheldrick, G. M. (2008). *Acta Cryst.* A**64**, 112–122. 10.1107/S0108767307043930 18156677

[bb5] Wu, C. D., Lu, C. Z., Chen, S. M., Zhuang, H. H. & Huang, J. S. (2003). *Polyhedron*, **22**, 3091–3098.

[bb6] Zhang, X. T., Dou, J. M., Wei, P. H., Li, D. C., Li, B., Shi, C. W. & Hu, B. (2009). *Inorg. Chim. Acta*, **362**, 3325–3332.

[bb7] Zhang, X. T., Wei, P. H., Sun, D. F., Ni, Z. H., Dou, J. M., Li, B., Shi, C. W. & Hu, B. (2009). *Cryst. Growth Des.* **9**, 4424–4428.

